# Correction: Zhou et al. The Positive Regulatory Effect of DBT on Lipid Metabolism in Postpartum Dairy Cows. *Metabolites* 2025, *15*, 58

**DOI:** 10.3390/metabo16020125

**Published:** 2026-02-12

**Authors:** Zheng Zhou, Kang Yong, Zhengzhong Luo, Zhenlong Du, Tao Zhou, Xiaoping Li, Xueping Yao, Liuhong Shen, Shumin Yu, Yixin Huang, Suizhong Cao

**Affiliations:** 1 Department of Clinical Veterinary Medicine, College of Veterinary Medicine, Sichuan Agricultural University, Chengdu 611130, China; fultz20zzheng@163.com (Z.Z.); zhengzhongluo@163.com (Z.L.); dzl960730@163.com (Z.D.); zhoutao0428@163.com (T.Z.); 13577@sicau.edu.cn (X.Y.); shenlh@sicau.edu.cn (L.S.); yayushumin@sicau.edu.cn (S.Y.); 2 Key Laboratory of Animal Disease and Human Health of Sichuan Province, College of Veterinary Medicine, Sichuan Agricultural University, Chengdu 611130, China; 3 Department of Animal Husbandry and Veterinary Medicine, College of Animal Science and Technology, Chongqing Three Gorges Vocational College, Chongqing 404105, China; yongkangkang@126.com; 4 Department of Clinical Veterinary Medicine, College of Veterinary Medicine, China Agricultural University, Beijing 100193, China; xiaopingli0512@163.com

The authors would like to make the following correction to their published paper [1]. The change is as follows:

## 1. Text Correction

In our previous publication, there was an error in the reference citation. Reference [11] in the original publication is incorrectly cited, and a correction has been made to Introduction, Paragraph 1:

Nutritional supplementation is an important strategy for maintaining metabolic homeostasis in dairy cattle during the peripartum period [11]. 

## 2. Reference

Reference [11] in the original publication is incorrectly cited and needs to be replaced with the following reference:

11. Gasselin, M.; Boutinaud, M.; Prézelin, A.; Debournoux, P.; Fargetton, M.; Mariani, E.; Zawadzki, J.; Kiefer, H.; Jammes, H. Effects of micronutrient supplementation on performance and epigenetic status in dairy cows. *Animal* **2020**, *14*, 2326–2335.

The authors state that the scientific conclusions are unaffected. This correction was approved by the Academic Editor. The original publication has also been updated.
