# Supplementary material for: The Positive Regulatory Effect of DBT on Lipid Metabolism in Postpartum Dairy Cows
Source: Metabolites. 2025 Jan 16;15(1):58. doi: 10.3390/metabo15010058 (PMC11767874; doi:10.3390/metabo15010058)
Supplement: Supplementary file 1 [file metabolites-15-00058-s001.zip › metabolites-3410413-supplementary.pdf]

Supplementary Material

1 Supplementary Methods

Targeted metabolomics analysis

Plasma sample (20 μL) were thoroughly mixed containing 120 μL of ice-cold methanol containing partial internal standards and then centrifuged at 4 °C (4000 g, 30 min). Supernatant (30 μL) was collected, derivative reagents (20 μL) were adde. The mixture was sealed, and derivatization was carried out at 30 °C for 60 min. After derivatisation, the sample was diluted with 330 μL of ice-cold 50% methanol solution, stored at -20 °C for 20 min and centrifuged at 4 °C (4000 g, 30 min). The supernatant (135 μL) was pipetted and 10 μL of internal standard was added. Serial dilutions of derivatised stock standards were added to the remaining wells. An ultra-performance liquid chromatography–tandem mass spectrometry (UPLC-MS/MS) system (ACQUITY UPLC-Xevo TQ-S, Waters Corp., Milford, MA, USA) was used to quantify all targeted metabolites. The system was equipped with an ACQUITY UPLC BEH C18 1.7 μM VanGuard pre-column (2.1 × 5 mm) and ACQUITY UPLC BEH C18 1.7 μM analytical column (2.1 × 100 mm), with an injection volume of 5 μL and flow rate of 0.4 mL/min. The column temperature was maintained at 40 °C. The mobile phase consisted of A (water with 0.1% formic acid) and B (acetonitrile/IPA; 70:30). The gradient elution procedure was as follows: 0–1 min (5% B), 1–5 min (5–30% B), 5–9 min (30–50% B), 9–11 min (50–78% B), 11–13.5 min (78–95% B), 13.5–14 min (95–100% B), 14–16 min (100% B), 16–16.1 min (100–5% B), and 16.1–18 min (5% B). The MS was alternately operated in positive-ion mode (+1.5 kV) and negative-ion mode (−2 kV). The source temperature was set to 150 °C, the desolvation temperature was set to 550 °C, and the desolvation gas flow was set to 1000 L/h. Raw data generated by UPLC-MS/MS were processed using MassLynx software (v4.1; Waters, Milford, MA, USA) for peak integration, calibration, and quantification of each metabolite. The iMAP platform (v1.0; MetaboProfile, Shanghai, China) was used for the statistical analyses. The concentrations of targeted metabolites were determined based on the calibration curves and corresponding regression coefficients.

2 Supplementary Tables

Supplementary Table S1 Ingredients and chemical composition of the diets for dairy cows

Item	DM basis, %	Item	DM basis, %
complete feeds	42.74	DM	49.91
fat powder	1	CP	18.48
molasses	3.76	NDF	30.5
corn silage	26.59	ADF	17.53
alfalfa hay	15.23	Ca	0.83
oat hay	2.32	P	0.45
beet pulp	3.57	NEL, Mcal/kg of DM	1.74
baking soda	0.25		
cottonseed	4.54		

Note: Guaranteed value (%) of composition of each kilogram of premix: crude protein≥24.8, crude fat≥4.66, crude fiber≤5.34, calcium≥0.92, phosphorus ≥0.4, lysine≥ 0.35, sodium chloride≥1. The net energy of lactation is the calculated value, and others are the measured value. DM=dry matter; CP= crude protein; NDF= neutral detergent fiber; ADF= acid detergent fiber; Ca= calcium;

P=phosphorus; NEL=net energy of lactation.

Supplementary Table S2 Dry matter intake in postpartum dairy cows

Item	Testing time	Group		
		Control	L-DBT	H-DBT
DMI	0d	7.17	7.04	7.21
(kg)	+7d	17.59	17.83	17.81
	+14d	18.03	18.09	18.13

Supplementary Table S3 Effect of DBT on the relative abundance of hindgut bacteria in dairy cows(+7d)

Item	Relative abundance		SEM	*P*-Value
	C7 (%)	DBT7 (%)		
UCG-009	1.070	0.664	0.092	0.038
Psychrobacillus	0	0.040	0.007	0.001
Bacillus	0	0.033	0.006	0.0001
Solibacillus	0	0.021	0.004	0.0002
Arthrobacter	0	0.019	0.003	0.006
Aerococcus	0	0.013	0.003	0.015

Supplementary Table S4 Effect of DBT on the relative abundance of hindgut bacteria in dairy cows(+14d)

Item	Relative abundance		SEM	*P*-Value
	C14 (%)	DBT14 (%)		
Paeniclostridium	6.573	3.286	0.655	0.009
Romboutsia	4.449	2.736	0.399	0.038
Alistipes	2.248	3.436	0.219	0.007
Clostridium_sensu_stricto_1	3.748	1.395	0.477	0.017
Dorea	0.091	0.246	0.026	0.001
Lachnospiraceae_UCG-010	0.051	0.174	0.034	0.028
Oscillibacter	0.031	0.142	0.023	0.004
Coprococcus	0.032	0.112	0.013	0.004
Phascolarctobacterium	0.031	0.108	0.022	0.023
Prevotellaceae_Ga6A1_group	0.018	0.044	0.005	0.012
Moryella	0.013	0.036	0.004	0.002

Negativibacillus	0.006	0.042	0.006	0.002
Lachnospira	0.028	0.002	0.008	0.045
Tuzzerella	0.004	0.019	0.003	0.015
Aeriscardovia	0.013	0.002	0.002	0.006
Gordonibacter	0.011	0.001	0.002	0.029
Hydrogenoanaerobacterium	0.0004	0.009	0.002	0.016
Eubacterium_xylanophilum_group	0.006	0	0.001	0.035

Supplementary Table S5 Differential metabolite screening of the C7 and DBT7 groups

Metabolites	C7(μmol/L)	DBT7(μmol/L)	Log2(FC)	*P*-value
Butyric acid	16.71±2.98	8.51±0.92	0.94	0.06
TCA	8.45±2.02	4.46±1.20	1.37	0.06
N-Acetylserotonin	0.48±0.05	0.62±0.04	-0.43	0.0003
2-Hydroxybutyric acid	14.64±1.37	11.04±0.99	0.41	0.05
Fumaric acid	0.62±0.10	1.30±0.21	-1.06	0.01
Malic acid	4.97±0.41	11.35±3.02	-0.89	0.001
3-Hydroxybutyric acid	582.65±47.07	449.47±22.11	0.37	0.02
Ethylmethylacetic acid	2.29±0.40	1.26±0.14	0.63	0.04
GABA	0.45±0.03	1.55±0.37	-1.10	0.002
Aspartic acid	4.93±0.33	5.77±0.22	-0.22	0.01
Ornithine	24.50±1.04	32.24±2.89	-0.39	0.02
Phenylacetylglutamine	0.59±0.03	0.55±0.04	0.18	0.06
Hydrocinnamic acid	10.62±0.92	8.25±0.53	0.37	0.04
2-Phenylpropionate	4.90±0.38	3.95±0.22	0.31	0.04
UCA	0.30±0.003	0.31±0.02	0.01	0.07
Acetylcarnitine	4.84±0.29	3.64±0.22	0.41	0.004

Note: Log2 (FC) > 0 (or < 0) indicated the metabolite concentration in C7 group were higher/lower than that in DBT7 group.

Supplementary Table S6 Differential metabolite screening of the C14 and DBT14 groups

Metabolites	C14(μmol/L)	DBT14(μmol/L)	Log2(FC)	*P*-value
GCA	25.78±3.49	17.52±2.15	0.56	0.06
GDCA	5.11±0.69	3.33±0.33	0.62	0.03

GCDCA	2.50±0.28	1.75±0.22	0.51	0.05
bUDCA	0.03±0.003	0.02±0.002	0.43	0.02
GUDCA	0.04±0.004	0.03±0.003	0.46	0.03
Citramalic acid	0.24±0.02	0.21±0.01	0.23	0.09
2-Hydroxy-3-methylbutyric acid	2.13±0.16	1.60±0.15	0.42	0.03
Suberic acid	1.03±0.06	0.88±0.06	0.23	0.08
Methylsuccinic acid	0.25±0.02	0.20±0.01	0.29	0.08
Asparagine	152.20±8.05	120.98±8.32	0.33	0.01
Serine	105.05±3.50	95.13±4.06	0.14	0.08
Mandelic acid	0.42±0.004	0.41±0.004	0.04	0.10
4-Aminohippuric acid	0.31±0.03	0.22±0.03	0.49	0.05
3-Hydroxyhippuric acid	0.54±0.09	0.38±0.02	0.12	0.04
Acetylcarnitine	4.54±0.44	3.48±0.19	0.33	0.06

Note: Log2 (FC) > 0 indicated the metabolite concentration in C14 group were higher than that in DBT14 group.

3 Supplementary Figures

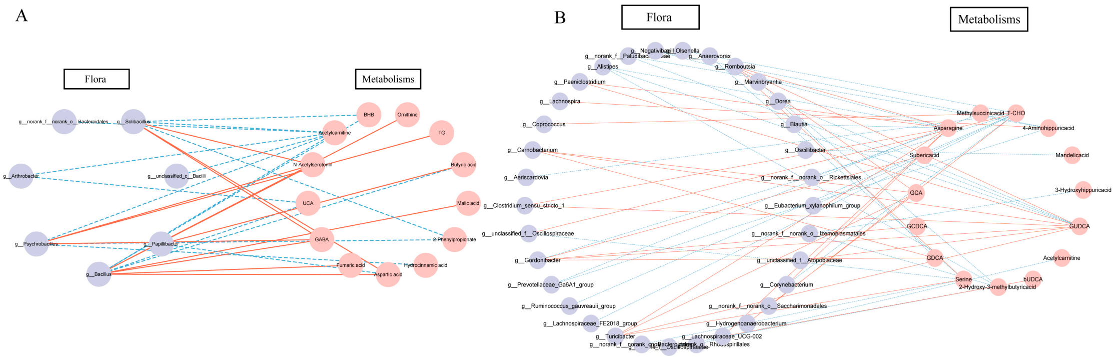

Supplementary Fig. S1 (A) correlation network diagram (+7d). The solid red line represents a positive correlation and the dashed blue line represents a negative correlation. (B) correlation network diagram (+14d).

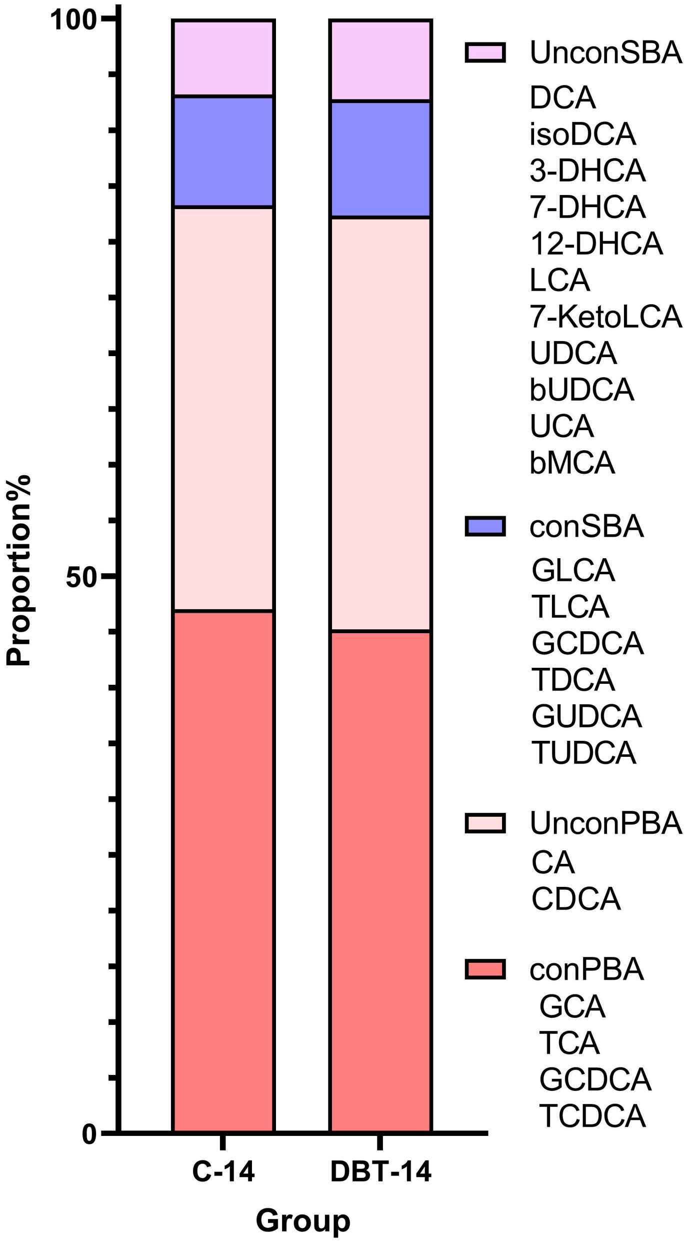

Supplementary Fig. S2 Histogram of the proportion of bile acids in plasma.
